# Supplementary material for: Nanocrystalline Cubic Phase Scandium-Stabilized Zirconia Thin Films
Source: Nanomaterials (Basel). 2024 Apr 18;14(8):708. doi: 10.3390/nano14080708 (PMC11054221; doi:10.3390/nano14080708)
Supplement: Supplementary file 1 [file nanomaterials-14-00708-s001.zip › nanomaterials-2940208-supplementary.pdf]

Supplementary Information Section

Substrate cleaning protocol

For basic cleaning, the Soda Lime Glass 0215 (Corning Glass, USA) and quartz (SPI Supplies, USA) substrates with the size of 75x25x1 mm were sonicated in a bath with Micro-90 cleaning solution (International Products Corporation, USA), diluted with distilled water 1:50, at 80°C during 10 min in an ultrasonic cleaner (GT Sonic-P3, China). The substrates were rinsed with deionized water to remove the residual cleaning solution and then kept in an ultrasonic bath with deionized water for 10 min. Further, the substrates were dried in airflow and sonicated in isopropanol for 10 min at 80°C. After that, the substrates were blown with dry nitrogen, immersed in boiling bi-distilled water (Milli-Q® IQ 7003, Merck, Germany) for 5 s, dried in a nitrogen flow, and finally heated in an oven (MRC MSF 11-4, Israel) for 20 min at 120°C. Clean substrates were stored in a desiccator under a vacuum. Before use, the glasses were cleaved into squares of 25x25 mm. Each substrate was rinsed in the spincoater (WS-650HZ-23NPPB, Laurell Technologies Inc, USA) subsequently with 3 ml bi-distilled water, 0.5 ml isopropanol, 3 ml bi-distilled water, 0.5 ml isopropanol, 3 ml bi-distilled water (x2 times) at 4000 rpm, and finally centrifuged at 6000 rpm for 15 s. After rinsing, glasses were dried in the oven for 20 min at 120°C. Just before the vacuum deposition, the substrates were processed in a plasma cleaner (MTI Corp. EQ-PCE3, USA) for 2 min at a “High” regime in residual air at 0.18 Torr.

The VST TSFP-842 sputtering chamber

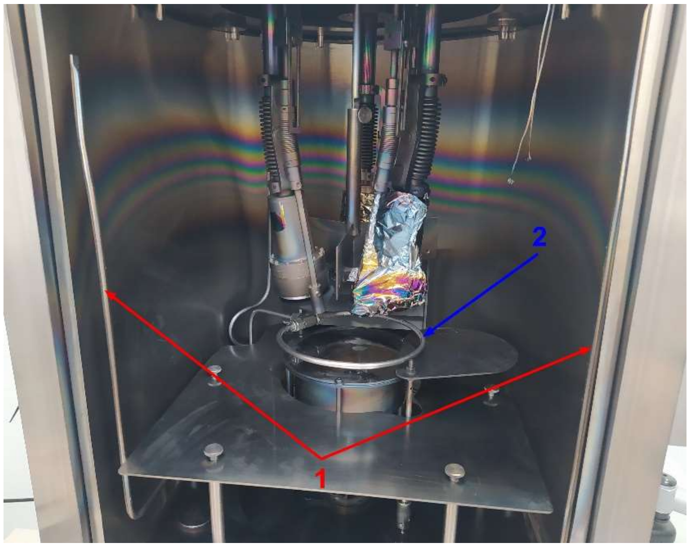

Figure S1. The working chamber of the VST TSFP-842 vacuum magnetron sputter. The red arrows point out the Ar supplying gas tubes. The blue arrow marks the reactive gas (O_2) tube.

The annealing thermograms of the ScSZ films

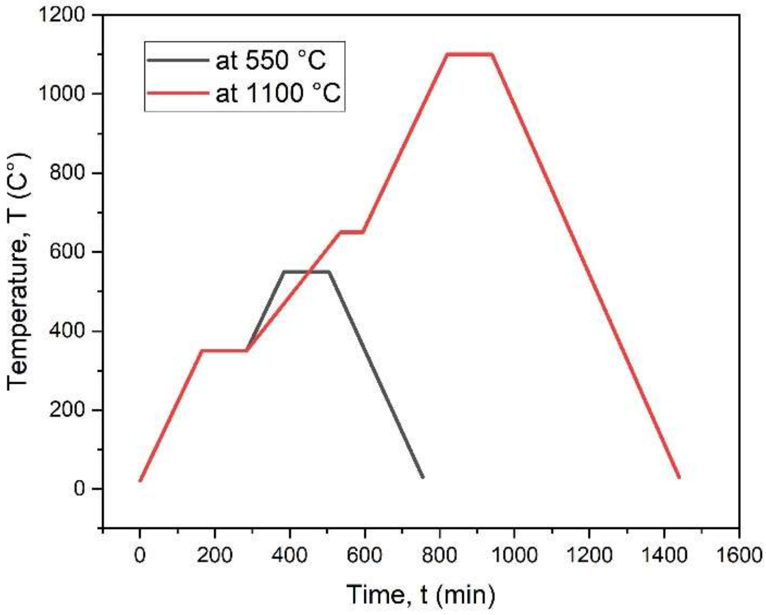

Figure S2. The annealing thermograms of the co-sputtered on the quartz substrates ScSZ films.

EDS and AES studying the ScSZ films

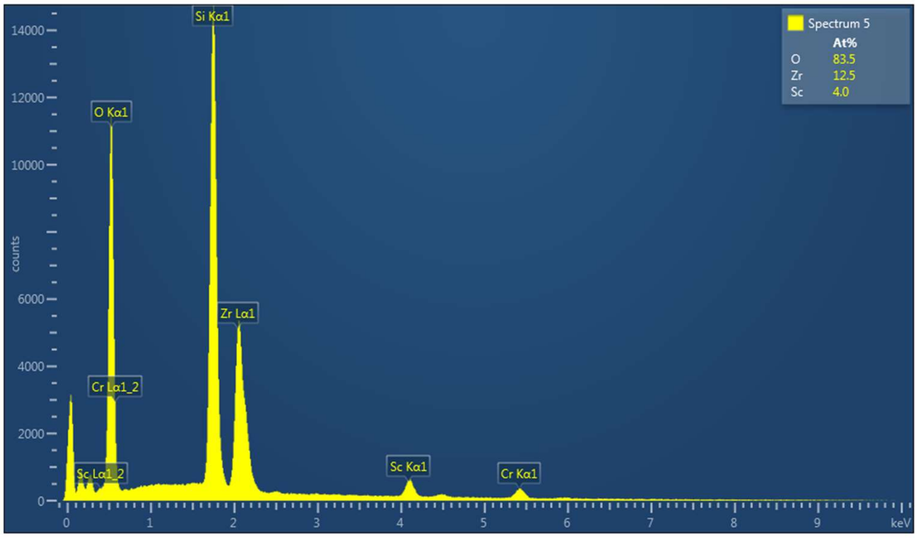

Figure S3. The EDS studying magnetron co-sputtered ScSZ films with 13.75 mol% of Sc_2O_3 content.

Prior to AES, the surface was treated with argon remote plasma as means to remove chemi- and physisorbed contaminants within a range of tens of nanometers of the surface. This was performed in a high vacuum chamber utilizing a downstream, inductively coupled RF-based system in which gas is supplied into the vacuum chamber through a needle leak-valve and a Pyrex tube inductively coupled with a Dressler Cesar 133 radio-frequency generator (Advanced Energy, USA). The direct plasma is generated inside the Pyrex tube as Ar gas is allowed to flow through at 2 sccm utilizing a MASS-FLO (MKS, USA) flow rate controller and excitated at 13.56 MHz. This plasma is then pulled towards the sample due to the pressure differential and additional application of potential to an electrode located underneath the sample stage with a Harrison 6448B DC power supply (Hewlett-Packard, USA). The remote plasma is then allowed to bombard the surface for 30 min to ensure thorough surface cleanliness. After this period, the system is brought to ~ 10^{-7} Torr and placed in the path of an EG3000 electron gun (LK Technologies, USA) operating at 3 KeV. The resulting Auger electrons are collected by a CMA2000 cylindrical mirror analyzer (LK Technologies, USA) over a range of 15 – 600 eV with a bias voltage of 1993 VDC and modulation amplitude of 10 V_{pp} at 2.5 kHz. The derivatives of the number of electrons are taken with respect to energy and plotted against Auger electron energy, as seen in fig. 4. From this spectrum, elemental concentrations are estimated from the intensity of the Auger transitions and elemental sensitivity. For Zr, a peak at ~147 eV associated with an MNN transition was utilized, whereas for Sc, an LMM transition centered at ~340 eV was used, and a KLL transition ~503 eV was utilized for O. Molecular concentrations are then reconstructed from the resulting atomic concentrations and tabulated.

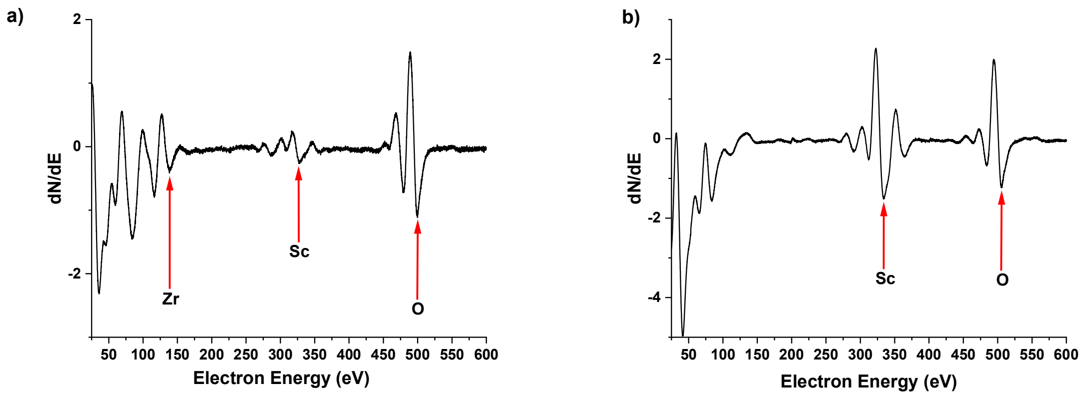

Figure S4. Differential Auger spectra for a) ScSZ thin film and b) Sc_2O_3 thin film with peaks utilized for elemental identification labeled.

Deposition rates of Zr and Sc

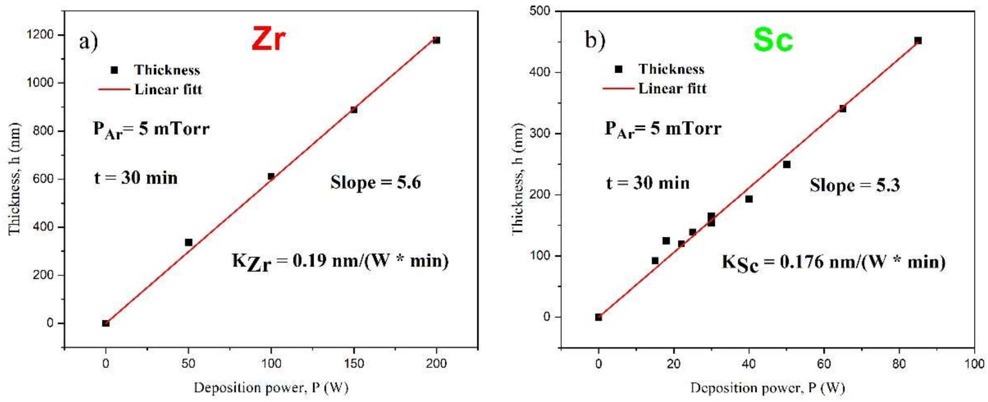

Figure S5. The deposition rates of the Zr and Sc sputtered films, a) and b), respectively.

Cluster approach

In the case of closed-packed regular SS, for the low concentration x of one component (***A***), its distribution in the matrix of the other (***B***) can be described within the so-called cluster approach [52]. The concentration of a single (one atom) "isolated" (surrounded only by ***B*** atoms) ***A*** clusters C_{1at} is expressed with formula (4). The expression (5) provides the exact concentration for clusters of a pair of ***A*** atoms C_{2at} (pairs clusters). The concentration of triples $\bar{C}_{3at}$ is determined from the relation (6). Note here that all ***A*** atoms in clusters with more than two atoms are accounted for only effectively, and C_{1at} and C_{2at} are exact expressions following the binominal distribution.

$$C_{1at} = x(1-x)^{12} \quad (4)$$

$$C_{2at} = 6x^2(1-x)^{18} \quad (5)$$

$$\bar{C}_{3at} = \frac{x-(C_{1at}+2C_{2at})}{3} \quad (6)$$

The relative concentrations of single, pair and triple clusters as a function of ***A*** concentration in the ***B*** matrix are presented in Figure S6.

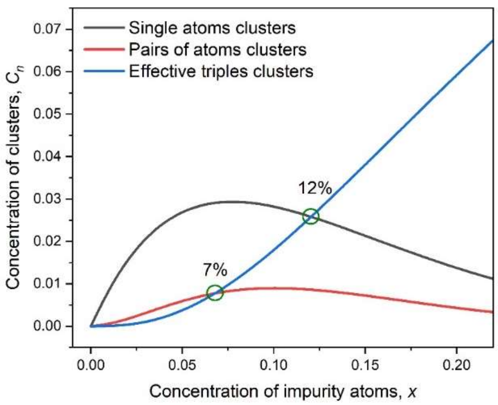

Figure S6. Relative concentrations of single, pair, and triple ***A*** clusters as a function of ***A*** atoms content in the fcc ***B*** matrix.

The analysis of the dependencies presented in Figure S6 shows that clusters of single atoms (black line) prevail in the region of low concentrations (up to 5 at%). Their number reaches a maximum at 7.7 at% of dopant and then decreases monotonously with increasing impurity concentration. The number of pair clusters (red line) becomes maximum at 10 at% but remains less than single ones. The amount of clusters with three or more atoms (blue line) becomes equal to pairs and singles at 7 and 12 at%, respectively, and continues to grow with increasing impurity concentration.
